# Unsupervised Wing-Bone Morphogroups in Bats Reveal Phylogenetic and Functional Patterns

**DOI:** 10.1093/iob/obag007

**Published:** 2026-03-09

**Authors:** S S Zalapa, J M Vargas-Aldaco, S Guerrero-Vázquez, F J López Chávez, E Ruiz-Sanchez

**Affiliations:** Departamento de Botánica y Zoología, Centro Universitario de Ciencias Biológicas y Agropecuarias, Universidad de Guadalajara, Camino Ramón Padilla Sánchez No. 2100, Las Agujas, 45200 Zapopan, Jalisco, México; Departamento de Botánica y Zoología, Centro Universitario de Ciencias Biológicas y Agropecuarias, Universidad de Guadalajara, Camino Ramón Padilla Sánchez No. 2100, Las Agujas, 45200 Zapopan, Jalisco, México; Departamento de Botánica y Zoología, Centro Universitario de Ciencias Biológicas y Agropecuarias, Universidad de Guadalajara, Camino Ramón Padilla Sánchez No. 2100, Las Agujas, 45200 Zapopan, Jalisco, México; Departamento de Botánica y Zoología, Centro Universitario de Ciencias Biológicas y Agropecuarias, Universidad de Guadalajara, Camino Ramón Padilla Sánchez No. 2100, Las Agujas, 45200 Zapopan, Jalisco, México; Departamento de Botánica y Zoología, Centro Universitario de Ciencias Biológicas y Agropecuarias, Universidad de Guadalajara, Camino Ramón Padilla Sánchez No. 2100, Las Agujas, 45200 Zapopan, Jalisco, México

## Abstract

Bat wings are complex biomechanical systems whose skeletal components play a central role in shaping flight performance. Although wing morphology in bats has traditionally been characterized using aerodynamic indices and ecological guilds, the contribution of individual wing bones to emergent patterns of wing design remains poorly understood. Here, we test whether interspecific variation in wing bone proportions is sufficient to generate objective, unsupervised morpho-wing bone groups (MWBGs) across Chiroptera. We quantified proportional variation in 10 wing bone elements relative to forearm length in 526 individuals representing 59 species from six bat families. Log-ratio-transformed measurements were analyzed using principal component analysis and hierarchical clustering, followed by linear discriminant analysis for group validation. We identified five robust MWBGs that explain coordinated variation in distal, intermediate, and proximal wing elements. These groups were recovered independently of *a priori* ecological classifications and showed higher discriminant performance than established foraging guilds. Phylogenetic mapping revealed that some MWBGs reflect strong phylogenetic conservatism (e.g., Molossidae), whereas others arise repeatedly across unrelated lineages, suggesting functional convergence in wing skeletal design. Distal phalanges and metacarpals emerged as key contributors to multivariate differentiation. Our results demonstrate that proportional variation in individual wing bones captures fundamental structural dimensions of bat wing morphology and provides a complementary, skeleton-centered framework for investigating the evolution of flight diversity in bats.

## Resumen

Las alas de los murciélagos son sistemas biomecánicos complejos cuyos componentes esqueléticos desempeñan un papel central en el rendimiento del vuelo. Aunque la morfología alar se ha descrito tradicionalmente mediante índices aerodinámicos y gremios ecológicos, la contribución específica de cada hueso del ala a los patrones emergentes de diseño alar sigue estando poco comprendida. En este estudio, evaluamos si la variación interespecífica en las proporciones de los huesos del ala es suficiente para generar grupos morfológicos de huesos alares objetivos y no supervisados dentro de Chiroptera. Cuantificamos la variación proporcional de diez elementos óseos en relación con la longitud del antebrazo en 526 individuos que representan 59 especies pertenecientes a seis familias. Las mediciones, transformadas en razones logarítmicas, fueron analizadas mediante análisis de componentes principales y agrupamiento jerárquico, y posteriormente validadas con un análisis discriminante lineal. Identificamos cinco grupos morfo-óseo alares (MWBGs) que explican variación coordinada en los elementos distales, intermedios y proximales del ala. Estos grupos, independientes de las clasificaciones ecológicas a priori, mostraron un rendimiento discriminante superior al de los gremios de forrajeo establecidos. El mapeo filogenético reveló que algunos MWBGs reflejan un fuerte conservadurismo filogenético (por ejemplo, en Molossidae), mientras que otros aparecen repetidamente en linajes no relacionados, lo que sugiere convergencia funcional en el diseño del esqueleto alar. Las falanges distales y los metacarpianos surgieron como elementos clave en la diferenciación multivariada. Nuestros resultados demuestran que la variación proporcional de los huesos individuales del ala captura dimensiones estructurales fundamentales de la morfología alar de los murciélagos y proporciona un marco complementario, centrado en el esqueleto, para investigar la evolución de la diversidad del vuelo en estos mamíferos.

## Introduction

Bat wings are composed of a variety of bones, joints, muscles, tendons, patagia, and sensory hair, which determine their shape and complex flight function (Swartz et al. 2007; Riskin et al. 2008; Sterbing-D’Angelo et al. 2011; Panyutina et al. 2015; Cheney et al. 2017). During flight, bats must counteract the drag and gravity imposed by physics by producing aerodynamic forces, such as thrust and lift (Norberg 2002; Swartz et al. 2012), while simultaneously responding to ecological and sensory challenges. Consequently, wing morphology reflects both shared physical constraints (Vaughan 1970) and variation that underlies differences in maneuverability, flight styles, and foraging strategies (Iriarte-Díaz and Swartz 2008; Bergou et al. 2015; Hedenström and Johansson 2015).

Wing bones shape the patagium, act as levers-arms for the muscles, resist deformations under aerodynamic loading (Swartz et al. 1992; Swartz et al. 2007), and are sufficiently light to optimize flight biomechanics (Cleland et al. 2021). However, the joint actions of bones, joints, muscles, and tendons modulate the 3D shape of the wing during flight to achieve maneuverability and the variety of flight behaviors that bats exhibit (Swartz et al. 2007; Riskin et al. 2008; Bahlman et al. 2016).

Lever mechanics predicts that the relative lengths of skeletal elements influence mechanical advantage (Dufour and Pillu 2018). If bat wing bones follow similar principles, interspecific variation in bone proportions should contribute to differences in flight function. This raises the question of whether proportional variation in wing bones can form distinct morpho-wing groups reflecting variation in wing form and performance.

Wing morphology has traditionally been described using wing loading, aspect ratio, and related indices (Farney and Fleharty 1969; Norberg and Rayner 1987). Despite known inconsistencies and incomplete taxonomic coverage, these indices remain widely used (Crane et al. 2022), and wing groups are commonly defined by combinations of high or low wing loading, aspect ratio, and wingtip shape (Norberg and Rayner 1987). Linear osteological measurements provide a complementary structural framework (Freeman 1981), yet they have rarely been explored comprehensively (Ševčík 2003; Panyutina et al. 2011). More recent studies have revisited bone-based approaches (Castillo-Figueroa 2020; Sánchez and Carrizo 2021; Gaudioso et al. 2023; Sánchez et al. 2025), but typically aggregate phalanges or omit certain digits. Geometric morphometrics offers another powerful analytical tool (Birch 1997; Schmieder et al. 2015); however, few studies explicitly evaluate the structural contribution of individual wing bones (Camargo and de Oliveira 2012; Magalhães de Oliveira et al. 2020; Ospina-Garcés et al. 2024; Argoitia et al. 2025). Additionally, bone contributions are commonly evaluated within *a priori* groupings defined by body size, wing indices, or ecological categories (Ševčík 2003; Panyutina et al. 2011; Castillo-Figueroa 2020; Sánchez and Carrizo 2021; Gaudioso et al. 2023; Ospina-Garcés et al. 2024) rather than emerging from skeletal variation itself. This pre-categorization constrains the capacity to isolate and interpret the functional contribution of individual wing elements.

Therefore, no study has systematically examined whether differences in individual wing bones are sufficient to generate emergent morpho-wing groups. Such an approach could contribute to our understanding of how structural variation contributes to functional diversity in flight.

A critical question is whether patterns of wing morphology are driven primarily by convergent functional adaptation or are largely conserved throughout their phylogenetic history. Comparative analyses indicate that many morphological and ecological traits in bats exhibit significant phylogenetic structure (Amador et al. 2020; López-Aguirre et al. 2021; Ospina-Garcés et al. 2024). Recent molecular phylogenies also provide robust hypotheses of evolutionary relationships across Chiroptera (Upham et al. 2019), offering a framework to disentangle phylogenetically conserved morphological patterns from those shaped by biomechanical or ecological convergence. Evaluating whether bone-based morpho-groups align with convergent functional adaptation or largely conserved throughout their phylogenetic history is therefore essential for interpreting the evolution of wing form.

In this study, we propose that interspecific variation in wing bone proportions gives rise to unsupervised MWBGs that capture structural aspects of wing design. Our objectives were to (1) quantify interspecific variation in the proportional length of each wing bone relative to forearm length, (2) identify emergent MWBGs using unsupervised multivariate methods, (3) evaluate their correspondence with classical functional wing categories, and (4) assess whether these groups are better explained by functional convergence or phylogenetic relatedness, using an independently derived species phylogeny.

## Methods

### Wing photographs

Wing photos were taken from two sources: (1) captured organisms and (2) individuals from the scientific collection of the Centro de Estudios en Zoología (collected between 2010 and 2020 by SSZ), Universidad de Guadalajara.

(1) Captured organisms: Bats were captured alive between 2015 and 2017 in Jalisco, Mexico. For each individual, standard measurements were taken, and species identification was subsequently determined. Photographs were taken using a Canon semiprofessional camera (PowerShot SX301S) mounted on a tripod and positioned horizontally above a millimetric cutting mat (Maped 15 × 30 cm, 1 × 1 cm grid). Each bat was gently placed in ventral recumbency. Two people held the bat still, holding its forearms and feet. The forearm and third digit of both wings were aligned with the grid line on the mat, allowing the fingers to stretch fully. Every photograph was taken for each individual without zoom, and all photos were cross-referenced with data recorded in the field book (SSZ).

The organisms were not harmed during the study, and restless individuals or pregnant females were released without being photographed. All handling of individuals followed the guidelines of the American Society of Mammalogists (Sikes and The Animal Care and Use Committee of the American Society of Mammalogists 2016). (2) Specimens from the scientific collection of the Centro de Estudios en Zoología de la Universidad de Guadalajara were also photographed, following the same process as for living organisms, provided that the epiphysis and bones were visible, regardless of whether the wing was folded. The catalog number was used as a reference for these specimens. For taxonomic classification purposes, we followed the nomenclature proposed by Simmons and  Cirranello (2025), except for *Artibeus intermedius*, which is considered synonymous with *A. lituratus*.

### Wing measurement

The photographs were processed using ImageJ software (Abramoff et al. 2004). Each photograph was independently calibrated using a grid mat. The epiphysis was identified from each pair of bones. Using the “measure” tool in ImageJ, eleven bone structures were evaluated from the right wing of each individual: forearm, metacarpus 2–5, and phalanges proximal and distal from digits 3 to 5 (Table 1). Each bone was measured according to its shape; for curved bones, measurements were taken in two or three segments (Freeman 1981).

**Table 1:** Wing bone structures: Labels and measurement definitions

Bone structure	Label	Description
Forearm	FA	Measurement of the total length of the radius; from the junction of the elbow to the wrist (carpus).
Metacarpal 2	MC2	Measure from the junction of the carpals to the end of the only phalanx.
Metacarpal 3	MC3	Measured from the junction of the carpals to the first epiphysis, on digit 3.
Phalanx proximal, digit 3	PP3	Measured from the first epiphysis to the second epiphysis, of digit 3.
Phalanx distal, digit 3	PD3	Measured from the second epiphysis to the end of digit 3.
Metacarpal 4	MC4	Measured from the junction of the carpals to the first epiphysis, at digit 4.
Phalanx proximal, digit 4	PP4	Measured from the first epiphysis to the second epiphysis, of digit 4.
Phalanx distal, digit 4	PD4	Measured from the second epiphysis to the end of the wing, of digit 4.
Metacarpal 5	MC5	Measured from the junction of the carpals to the first epiphysis, at digit 5.
Phalanx proximal, digit 5	PP5	Measured from the first epiphysis to the second epiphysis, of digit 5.
Phalanx distal, digit 5	PD5	Measured from the second epiphysis to the end of the wing, digit 5.

### Data analysis

We considered the second phalanx and the distal phalanx of digits 3–5 as a single variable (PD) because the distal phalanx varies between species (absent, small, cartilaginous, or ossified; Bahlman et al 2016; Gaudioso 2019). All measurements were transformed using the natural logarithm (ln) to stabilize variance and approximate linear allometric relationships. Subsequently, logarithmic ratios were calculated between each bone (*y*) and forearm (*x*) as reference structure, expressed as ln(*y*) − ln(*x*), in order to remove absolute scale effects and robustly capture relative body proportions (Mosimann 1970; Aitchison 1986). For interpretative purposes, log-ratio values were back-transformed using the exponential function $({e^x} = {e^{{\mathrm{ln}} ( y ) - {\mathrm{ln}} ( x )}})$ and proportional deviations from the forearm were expressed as percentages (calculated as $({e^x} - 1)\,\, \times \,\,100)).$ We chose forearm length as the reference structural measure, as it shows an isometric relationship with body mass. Previously we quantified its scaling relationship with body mass, using a Type II standardized major axis (SMA) regression (Jolicoeur 1963; Warton et al. 2006). We included body mass data from 510 individuals (representing 55 species) of the 526 individuals in the morphometric analysis. The estimated slope was *b* = 2.989, with a 95% confidence interval of 2.905–3.076. SMA provides an appropriate functional estimate of morphological scaling when the primary goal is to characterize the covariation between traits rather than to predict one variable from another (Smith 2009). To test for isometry, we compared the estimated SMA slope and its 95% confidence interval with the theoretical expectation for mass-length scaling (*b* = 3).

Principal component analysis (PCA) was performed on the correlation matrix, with all variables standardized to unit variance prior to analysis. The use of a correlation-based PCA is appropriate for exploratory multivariate analyses when variables, although expressed on the same logarithmic scale, differ in their variances or levels of dispersion (Jolliffe 2002). Component retention was assessed using the broken-stick criterion as an objective stopping rule (Jackson 1993). For the interpretation of the retained components, a threshold ≥0.50 for factor loadings was adopted, a criterion widely used in studies that follow the methodological guidelines of Hair et al. (2019). The morphometric centroid for each species was calculated by averaging the PCA correlation scores of each individual across the first three principal components, thereby reducing the influence of unequal sample sizes among species and allowing each taxon to be represented as a single mean point within the morphospace (Dryden and Mardia 2016). This strategy is particularly suitable when the aim is to explore emergent groupings without imposing prior hypotheses. Although some species were represented by only one or a few individuals, they were not excluded from the analysis. This decision reflects the fact that the purpose of the study was not to assess intraspecific variability, but rather to position each species as an average unit within the multivariate morphological space, following precedents in exploratory comparative analyses (Adams and Collyer 2019). We applied hierarchical clustering using Ward’s minimum-variance method, to explore structural patterns among the 59 species. This agglomerative criterion merges clusters by minimizing the increase in the total within-cluster sum of squares at each step, thereby producing compact and internally homogeneous groups (Ward 1963). Because our variables represent standardized correlations between skeletal elements and the principal components, quantifying the contribution of each structure to the multivariate morphological gradients, this metric provides a consistent basis for comparing species in a Euclidean space. Ward’s method is explicitly designed for interval-scaled data and Euclidean distances, and it has been shown to yield stable partitions under these conditions (Kaufman and Rousseeuw 1990). Accordingly, it is well suited to morphometric applications in which the goal is to identify clusters defined by reduced within-group variance. PCA and CA was conducted using PC-ORD v7.11 (McCune and Mefford 2018).

We conducted a linear discriminant analysis (LDA) to evaluate the robustness of the emergent clusters obtained from the exploratory analyses using logarithmic ratios each bone for all 526 individuals rather than species-level centroids. This decision was based on the requirement of LDA for intraspecific variance to estimate discriminant boundaries and to quantify the reclassification accuracy of the model-conditions that cannot be met when only a single mean value per species is used (Huberty and Olejnik 2006). The categorical variable for this first LDA corresponded to the groups defined by the hierarchical clustering analysis. To further assess whether the emergent morpho-wing bone clusters reflect functional differentiation, we repeated the LDA using the foraging guilds categories proposed by Ospina-Garcés et al. (2024; Table S1) as the grouping variable. This complementary analysis provided a comparative benchmark to evaluate the discriminant performance of bone-based groupings relative to an established functional classification. The predictive power of the discriminant model was tested by the cross-validation method (McLachlan 2004). LDA was performed with MASS v7.3–65 (Venables and Ripley 2002) in R Suite (R Core Team 2021).

The phylogenetic tree was pruned from a set of 100 mammalian time-calibrated trees (node-dated, containing 5911 species) from Upham et al. (2019), which were downloaded from http://vertlife.org/phylosubsets. Subsequently, we generated a maximum clade credibility tree using Mesquite v. 4.02 (Maddison and Maddison 2025).

## Results

We analyzed the proportional length of 10 wing bone measurements from 526 individuals from 59 species (Table S1), representing six families and 37 genera. Phyllostomidae was the most abundant family (25 species), followed by Vespertilionidae (20 species), Molossidae (eight species), Mormoopidae (three species), Emballonuridae (two species), and Natalidae (one species).

**Fig. 1:**
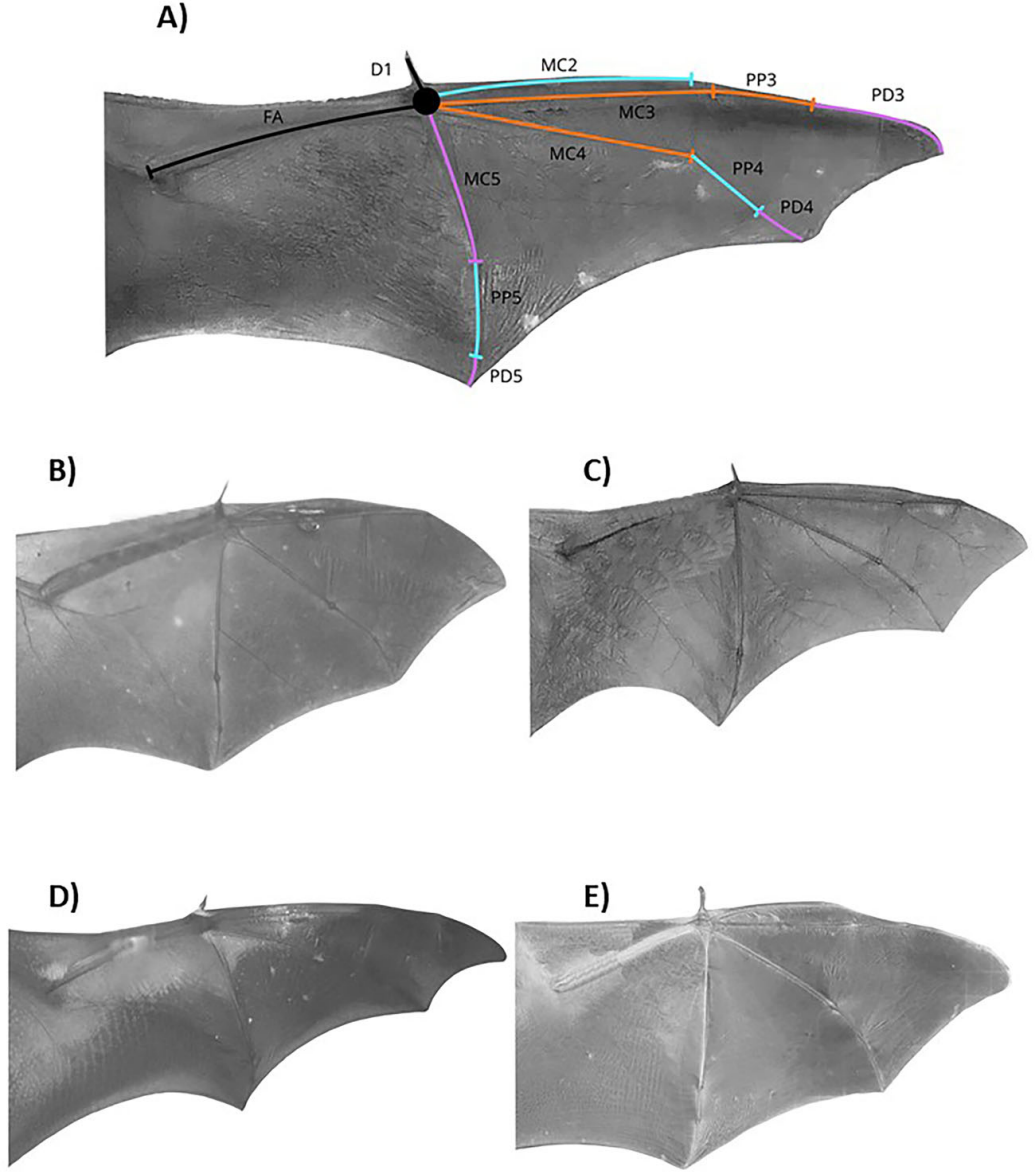
Wing morphology of each MWBG: (A) MWBG1, (B) MWBG2, (C) MWBG3, (D) MWBG4, and (E) MWBG5. In panel (A), wing bones are color-coded according to their principal component on which each exhibits its highest loading.

The PCA revealed strong multivariate structure in bat wing morphology. The first three components accounted for 81.47% of the total variance, with PC1 explaining 36.80%, PC2 explaining 26.66%, and PC3 explaining 18.01%. Only these three components exceeded the variance expected under the broken-stick model, indicating that they represent biologically meaningful axes of variation. PC1 was characterized by high positive loadings on PD4 (0.8969), PD5 (0.8414), MC5 (0.7886), and PD3 (0.7801) and negative loading on PP5 (−0.5883). PC2 was dominated by high negative loadings on MC4 (−0.8549), MC3 (−0.784), PP3 (−0.5979), and MC2 (−0.5764). PC3 was dominated by high positive loading on MC2 (0.6113), and negative loadings on PP4 (–0.6835) and PP5 (–0.6647) (Table S2, Fig. 1A).

**Fig. 2:**
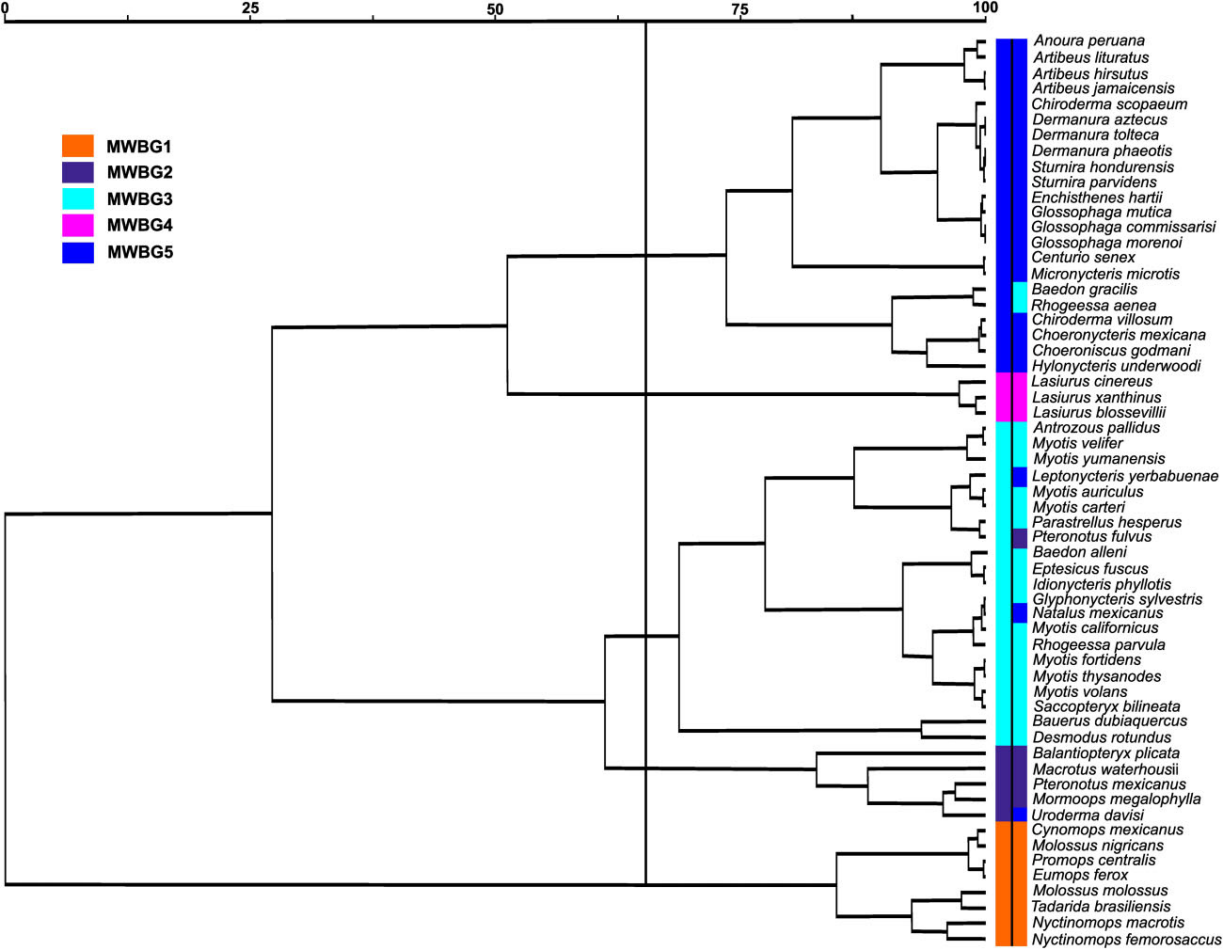
Dendrogram derived from cluster analysis, including 59 species representing six families and 37 genera. Five major groups were partitioned at approximately 65% information remaining (black line; left color column). The dendrogram was reconstructed by incorporating cross-validated reclassification results from the LDA, thereby reflecting corrected species cluster membership (right color column). MWBG, morpho-wing bone group.

Hierarchical clustering of proportional wing‐bone measurements identified five major groups when the dendrogram was partitioned at approximately 65% information remaining. The resulting dendrogram shows a clear segregation among clusters, with morphometric similarity progressively decreasing toward the right side of the plot (Fig. 2). LDA applied to the groups derived from hierarchical clustering revealed a highly robust discriminant structure. The first canonical axis explained 69.2% of the total variance, followed by 18.8% on the second axis, indicating that most of the multivariate differentiation among groups is captured within a low‐dimensional canonical space. Classification performance was high (overall accuracy = 98.29%; LOOCV = 97.72%), with misclassifications limited to a small number of individuals in three groups, while the remaining groups exhibited nearly perfect reclassification (Table S3A; Fig. S1-A). Finally, the dendrogram was reconstructed by incorporating the cross‐validated reclassification results from the discriminant analysis, thereby reflecting the corrected cluster membership of species (Fig. 2).

When the LDA was repeated using the foraging guilds categories proposed by Ospina-Garcés et al. (2024) as the grouping variable, the discriminant performance declined (direct classification = 91.63%; LOOCV = 90.3%). The lowest-performing group was the edge-space aerial forager group (70.83%), followed by the open-space aerial forager (92%). The narrow-space gleaning foragers had 98.8% accuracy, and only the edge-space trawling foragers achieved 100%. Of the 44 individuals originally lacking functional assignments, 11 remained unclassified (Table S3B, Fig. S1-B).

Five MWBGs were identified. These groups were characterized by specific combinations of the proportional bone lengths (classified as relatively high, intermediate, or low compared with other groups) according to the magnitude (greater, moderate, or lesser) of percentages reductions relative of forearm length.

Morpho-wing bone group 1 (MWBG1): This group includes insectivorous species of Molossidae: *Cynomops mexicanus, Eumops ferox, Molossus molossus, Molossus nigricans, Nyctinomops femorosaccus, Nyctinomops macrotis, Promops centralis*, and *Tadarida brasiliensis*. Bones associated with PC1 exhibited the widest proportional lengths relative to the forearm, with the greatest reductions observed in PD4 (−85.9%), PD5 (−87.0%), MC5 (−42.7%), PD3 (−52.1%), and the smallest reduction in PP5 (−71.5%). In bones associated with PC2 showed low-to-high proportional lengths, including MC4 (−4.2%), MC3 (−1.6%), PP3 (−62.4%) and MC2 (−11.8%). Bones loading on PC3 similarly displayed low-to-high proportional lengths, represented by MC2 (−11.8%), PP4 (−69.2%), and PP5 (−71.5%) (Table 2; Fig. 1A).

**Table 2:** Back-transformed logarithmic ratios of wing bone structures for each MWBG

	PC1				PC2			PC3		
Group	PD4	PD5	MC5	PD3	MC4	MC3	PP3	MC2*	PP4	PP5*
MWBG1	0.14	0.13	0.57	0.48	0.96	0.98	0.38	0.88	0.31	0.28
(*n* = 26)	(0.06–0.22)	(0.07–0.17)	(0.49–0.68)	(0.41–0.52)	(0.89–1.07)	(0.92–1.06)	(0.21–0.48)	(0.72–0.99)	(0.20–0.40)	(0.23–0.35)
*P*	−85.9	−87	−42.7	−52.1	−4.2	−1.6	−62.4	−11.8	−69.2	−71.5
MWBG2	0.2	0.2	0.72	0.56	0.76	0.82	0.23	0.79	0.22	0.25
(*n* = 8)	(0.09–0.27)	(0.12–0.24)	(0.64–0.78)	(0.36–0.69)	(0.69–0.84)	(0.73–0.96)	(0.15–0.32)	(0.69–0.90)	(0.18–0.28)	(0.19–0.28)
*P*	−80.2	−80.4	−28.4	−44.0	−23.6	−17.8	−77.5	−21.4	−77.8	−75.4
MWBG3	0.26	0.19	0.88	0.45	0.9	0.92	0.31	0.88	0.25	0.22
(*n* = 34)	(0.20–0.34)	(0.12–0.26)	(0.69–0.98)	(0.29–0.63)	(0.84–0.99)	(0.85–0.99)	(0.17–0.42)	(0.68–1.10)	(0.14–0.35)	(0.14–0.32)
*P*	−73.8	−81.2	−11.5	−55.2	−9.8	−8.0	−68.5	−12.3	−75.2	−78.0
MWBG4	0.26	0.19	0.85	0.47	1.01	1.1	0.36	1.3	0.24	0.16
(*n* = 14)	(0.20–0.30)	(0.17–0.24)	(0.75–0.97)	(0.40–0.53)	(0.94–1.04)	(1.03–1.47)	(0.30–0.40)	(1.23–1.40)	(0.19–0.28)	(0.14–0.21)
*P*	−73.9	−81.1	−15.1	−52.9	0.6	10.4	−64.2	29.5	−76.3	−83.8
MWBG5	0.35	0.29	0.9	0.79	0.92	0.93	0.32	0.87	0.26	0.21
(*n* = 444)	(0.25–0.43)	(0.20–0.40)	(0.77–1.04)	(0.58–0.98)	(0.74–1.05)	(0.77–1.12)	(0.23–0.45)	(0.64–1.06)	(0.21–0.33)	(0.16–0.33)
*P*	−65.5	−70.6	−10.1	−20.7	−8.2	−6.6	−68.1	−13.0	−73.8	−78.8

*Note: n* = number of individuals. Values are presented as mean (min–max). *P*, percentage of proportional deviation from the forearm (calculated as $[{e^x} - 1] \times 100)$, representing the percentage reductions relative of forearm length. Variables are organized according to their strongest principal component (PC) loadings. *Variables with high loadings on two components: PP5 on PC3 and PC1; MC2 on PC3 and PC2.

Morpho-wing bone group 2 (MWBG2): This group encompasses insectivorous species of Mormoopidae: *Mormoops megalophylla, Pteronotus fulvus*, Emballonuridae: *Balantiopteryx plicata*; and insectivorous of Phyllostomidae: *Macrotus waterhousii*. Bones associated with PC1 exhibited intermediate proportional lengths relative to the forearm, with moderate reductions in PD4 (−80.2%), PD5 (−80.4%), MC5 (−28.4%), PD3 (−44.0%), and PP5 (−75.4%). In contrast, bones associated with PC2 showed the lowest proportional lengths, represented by MC4 (−23.6%), MC3 (−17.8%), PP3 (−77.5%), and MC2 (−75.4%). Bones loading on PC3 displayed low-to-intermediate proportional lengths, including MC2 (−21.4%), PP4 (−77.8%), and PP5 (−75.4%) (Table 2; Fig. 1B).

Morpho-wing bone group 3 (MWBG3): This group includes insectivorous species of Vespertilionidae: *Antrozous pallidus, Baeodon alleni, Baeodon gracilis, Bauerus dubiaquercus, Eptesicus fuscus, Idionycteris phyllotis, Myotis auriculus, Myotis californicus, Myotis carteri, Myotis fortidens, Myotis thysanodes, Myotis velifer, Myotis volans, Myotis yumanensis, Parastrellus hesperus, Pteronotus mexicanus, Rhogeessa aenea, Rhogeessa parvula*; Emballonuridae: *Saccopteryx bilineata*; and Phyllostomidae: *Desmodus rotundus*. Bones associated with PC1 exhibited a heterogeneous pattern of proportional contributions, spanning relatively high, intermediate, and low proportional lengths, with greater, moderate, and lesser reduction observed in PD4 (−73.8%), PD5 (−81.2%), MC5 (−11.5%), PD3 (−55.2%), and PP5 (−78.0%). In contrast, bones associated with PC2 displayed intermediate-to-high proportional lengths, represented by MC4 (−9.8%), MC3 (−8.0%), PP3 (−68.5%), and MC2 (−12.3%). Bones loading on PC3 similarly showed intermediate-to-high lengths, including MC2 (−12.3%), PP4 (−75.2%), and PP5 (−78.0%) (Table 2; Fig. 1C).

Morpho-wing bone group 4 (MWBG4): This group includes insectivorous species of Vespertilionidae: Vespertilioninae: Lasiurini: *Lasiurus cinereus, Lasiurus frantzii*, and *Lasiurus xanthinus*. Bones associated with PC1 exhibited high, intermediate, and low proportional lengths, with greater, moderate, and lesser reductions including PD4 (−73.9%), PD5 (−81.1%), MC5 (−15.1%), PD3 (−52.9%), and PP5 (−83.8%). In contrast, bones associated with PC2 showed the highest proportional lengths, represented by MC4 (0.6%), MC3 (10.4%), PP3 (−64.2%), and MC2 (29.5%). Bones loading on PC3 displayed high-to-low range of proportional lengths, including MC2 (29.5%), PP4 (−76.3%), and PP5 (−83.8%) (Table 2; Fig. 1D).

Morpho-wing bone group 5 (MWBG5): This group includes species of Phyllostomidae: Nectarivorous Glossophaginae: *Anoura peruana, Choeroniscus godmani, Choeronycteris mexicana, Glossophaga commissarisi, Glossophaga morenoi, Glossophaga mutica, Hylonycteris underwoodi*, and *Leptonycteris yerbabuenae*; Frugivorous Stenodermatinae: *Artibeus hirsutus, Artibeus jamaicensis, Artibeus lituratus, Chiroderma scopaeum, Chiroderma villosum, Centurio senex, Dermanura azteca, Dermanura phaeotis, Dermanura tolteca, Enchistenes hartii, Sturnira hondurensis, Sturnira parvidens*, and *Uroderma davisi*; Insectivorous Glyphonycterinae: *Glyphonycteris sylvestris*, Micronycterinae: *Micronycteris microtis* and *Natalus mexicanus* (Natalidae). Bones associated with PC1 exhibited the highest proportional lengths relative to the forearm, with the lowest reductions observed in PD4 (−65.5%), PD5 (−70.6%), MC5 (−10.1%), PD3 (−20.7%), and PP5 (−78.8%). In contrast, bones associated with PC2 displayed intermediate to high proportional lengths, represented by MC4 (−8.2%), MC3 (−6.6%), PP3 (−68.1%), and MC2 (−13.0%). Bones loading on PC3 showed low-to-intermediate proportional lengths, including MC2 (−13.0%), PP4 (−73.8%), and PP5 (−78.8%) (Table 2; Fig. 1E).

**Fig. 3:**
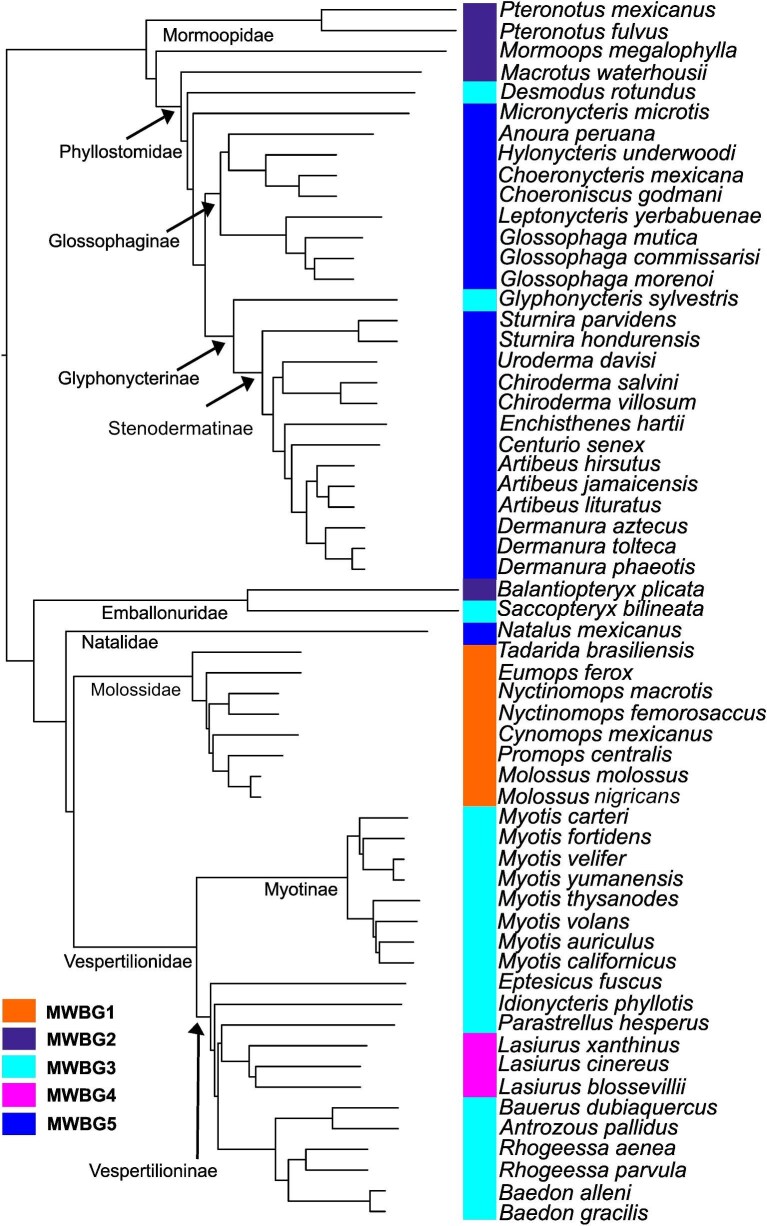
Phylogenetic relationships among sampled taxa based on the time-calibrated phylogeny of Upham et al. (2019). Colored bars denote species assignment to each MWBG.

Our maximum clade credibility phylogeny provides an evolutionary context for the five MWBGs. Within this tree, all families are monophyletic except Mormoopidae. The phylogenetic distribution of the groups is as follows: MWBG1, all Molossids; MWBG2, *Pteronotus fulvus* + *Pteronotus mexicanus* + *Mormoops megalophyla* + *Balantiopteryx plicata* + *Macrotus waterhousii*; MWBG3, most Vespertilionidae + *Saccopteryx bilineata* + *Desmodus rotundus*; MWBG4, all *Lasiurus*; and MWBG5, almost all Phyllostomidae + *Natalus mexicanus* (Fig. 3).

## Discussion

Our analysis shows that proportional differences in individual wing bones define five emergent MWBGs that reflect a combination of phylogenetic, aerodynamic specialization, and ecological strategies constraints. These clusters were recovered from unsupervised multivariate methods and validated by discriminant reclassification, indicating that variation in bone proportions can retain coordinated functional signals (Ševčík 2003; López-Aguirre et al. 2021; Gaudioso et al. 2023).

### Morpho-wing bone groups

Patterns of proportional bone variation recovered in the MWBGs are consistent with known kinematic and aerodynamic differences among bat flight modes. Experimental studies demonstrate that wing kinematics are not uniform across Chiroptera; rather, species differentially modulate joint excursions, aeroelastic deformation, camber, and spanwise twist according to body plan, ecological context, and aerodynamic demands (Iriarte-Díaz and Swartz 2008; Hubel et al. 2012, 2016; von Busse et al. 2012; Hedenström and Johansson 2015). These kinematic adjustments are accompanied by additional structural and functional features that also vary among species, including differences in distal phalangeal morphology and metacarpophalangeal joint configuration (Bahlman et al. 2016), elastin fiber architecture and intramembranous musculature within the wing membrane (Cheney et al. 2017), skeletal specializations in the humerus and radius (Sánchez et al. 2025), and behavioral traits such as landing mechanics (Boerma and Swartz 2024).

Species assigned to MWBG1 (Fig. 1A), corresponding to Molossidae, are defined by proportionally shortened distal phalanges and a reduced fifth metacarpal (MC5). This configuration is consistently associated with high to near-average aspect ratios and elevated wing loading, aerodynamic conditions that favor high minimum flight speeds and reduced energetic costs during sustained forward flight (Norberg and Rayner 1987; Iriarte-Díaz et al. 2002). Functional insight into this morphotype is provided by studies of *T. brasiliensis*, which exhibits a nearly vertical stroke plane and largely planar wing motion, with restricted distal joint excursions and an absence of pronounced digital curling (Hubel et al. 2012). These kinematic patterns are consistent with the presence of proportionally short, fully ossified terminal phalanges (this study; Bahlman et al. 2016), which likely limit fine-scale deformation of the distal wing surface. The functional consequences of this distal rigidity extend beyond flight, as *T. brasiliensis* employs minimally rotational, high-impact landing strategies (Boerma and Swartz 2024), suggesting a reduced capacity to dissipate kinetic energy through distal wing deformation. Together with simplified intramembranous musculature (Cheney et al. 2017), these traits indicate a generally rigid and stable wing-body configuration optimized for fast, sustained flight in open environments. Importantly, this apparent rigidity does not imply mechanical simplicity: molossids retain a relatively high number of fully actuated joints, enabling active modulation of wing shape sufficient for capturing mobile prey in open air (Bahlman et al. 2016). In addition, reduced deltopectoral crests and other molossid-specific skeletal traits are associated with quadrupedal locomotion (Sánchez et al. 2025), underscoring functional trade-offs that extend beyond aerial performance. Despite the overall consistency of this morphotype, recent analyses have revealed intra-group variation in wing shape and size within Molossidae (Argoitia et al. 2025), indicating that even highly specialized wing configurations retain capacity for fine-scale functional tuning.

In contrast, MWBG2 (Fig. 1B) encompasses a morphologically convergent assemblage including almost all mormoopids, a subset of emballonurids, and *M. waterhousii*, which share moderately developed distal elements and exhibits the shortest MC3, MC4, PP3, and MC2 (Table 2) despite substantial phylogenetic distance. Aerodynamically, these species occupy an intermediate regime, with aspect ratios higher than those of most phyllostomids and lower wing loading than molossids. Within this shared proportional framework, however, flight behavior varies markedly, ranging from the rapid, sustained yet maneuverable flight of mormoopids near surfaces to the slower, erratic but highly maneuverable flight of *B. plicata* (Norberg and Rayner 1987). Comparative analyses of wing soft tissues reveal both similarities and differences in the organization of elastin bundles and intramembranous musculature between Emballonuridae and Mormoopidae (Cheney et al. 2017), suggesting that similar skeletal proportions can be supported by different material architectures. Skeletal variation within this group further illustrates how distinct functional solutions may arise within a common morphospace. For example, *Pt. davyi* and *Mo. megalophylla* differ in distal phalangeal ossification and in the number of fully actuated joints, indicating different balances between active control and passive deformation of the wing (Bahlman et al. 2016). Notably, this capacity for in-flight modulation contrasts with landing behavior in species such as *Pt. fulvus*, which employ minimally rotational, high-impact landings (Boerma and Swartz 2024), reinforcing the idea that the mechanical demands of flight and landing may be partially decoupled.

MWBG3 (Fig. 1C) represents a further shift toward functional versatility, combining an elongated MC5 with a relatively short distal phalanx of digit 3 and MC2, and intermediate proportions in the remaining wing elements (Table 2). This morphotype, dominated by vespertilionids, spans a wide range of wing loading values but is generally associated with average to low aspect ratios, a combination typically linked to slow and maneuverable flight (Norberg and Rayner 1987). Kinematic studies of *My. velifer* and *My. lucifugus* demonstrate how this skeletal configuration enables extensive modulation of wingbeat frequency, stroke amplitude, and stroke-plane orientation to maintain aerodynamic force balance across a broad range of flight speeds and environmental contexts (Aldridge 1988; Hubel et al. 2016). This kinematic flexibility is mirrored by substantial skeletal diversity within MWBG3, including variation in distal element ossification, the presence or absence of ventral tendons, and the number of fully and hemi-actuated joints (Bahlman et al. 2016). Despite this versatility in flight, landing mechanics in several vespertilionids converge on minimally rotational, high-impact strategies similar to those observed in MWBG1, whereas *S. bilineata* exhibits highly rotational, low-impact landings (Boerma and Swartz 2024). This pattern highlights the multifunctional nature of the wing in this group and further supports a decoupling between in-flight maneuverability and landing performance.

MWBG4 (Fig. 1D), unique to *Lasiurus*, exhibits a distinct morphotype characterized by pronounced elongation of MC4, MC3, MC2, and PP3, indicating a strong emphasis on proximal wing elements. *La. cinereus* combines high aspect ratio with low wing loading, a configuration classically associated with fast, stable flight in open spaces and limited maneuverability (Norberg and Rayner 1987). However, the scarcity of detailed kinematic, anatomical, and behavioral data currently limits functional interpretation of this consistently distinctive morphotype, highlighting an important gap for future integrative studies.

Finally, MWBG5 (Fig. 1E) displays the opposite osteological pattern to MWBG1, with elongated distal wing elements (PD4, PD5, PD3, and MC5; Table 2) and a broad spectrum of ecological and functional strategies. This group includes nectarivorous, frugivorous, and insectivorous phyllostomids, as well as natalids, and spans flight modes ranging from slow flight and hovering to highly maneuverable flight in cluttered environments (Norberg and Rayner 1987). This ecological breadth is reflected in wide variation in wing loading and generally low to average aspect ratios (Norberg and Rayner 1987). Although species such as *L. yerbabuenae* and *G. soricina* generate broadly similar aerodynamic force patterns, differences in force timing and distribution across the wingbeat cycle reveal distinct functional solutions within a shared morphospace (Muijres et al. 2011; von Busse et al. 2012). Structurally, shared traits such as cartilaginous distal phalangeal tips, combined with variation in ventral tendons and joint actuation, suggest differing degrees of passive, aerodynamically induced deformation vs. active control of wing shape (Bahlman et al. 2016). The complex wing architecture of *Ar. jamaicensis*, including dense elastin networks and intramembranous musculature (Cheney et al. 2017), together with proximal skeletal traits associated with increased joint stability in *Ar. lituratus* (Sánchez et al. 2025), further underscores the functional diversity of this group. Consistent with these structural differences, landing strategies in MWBG5 range from low- to high-impact contacts and variable body rotations (Boerma and Swartz 2024).

Despite this growing body of evidence, integrative studies explicitly evaluating the kinematic interactions among wing components and the functional and ecological demands imposed on the wing remain scarce. Although our osteological analysis recovered robust MWBGs, species within MWBGs 2, 3, and 5, exhibited substantial variation in wing shape (Norberg and Rayner 1987), structural traits (Bahlman et al. 2016; Cheney et al. 2017), and behavioral strategies (Boerma and Swartz 2024). This pattern indicates that similar wing shapes, aerodynamic performance, and flight modes are not necessarily achieved through identical skeletal proportions. Consequently, our results raise the key questions: Are comparable functional demands met through different structural configurations of the wing? Addressing this question will require objective, integrative studies explicitly linking skeletal structure, kinematics, aerodynamic performance, and ecology.

### MWBGs vs. foraging guilds

Compared with the foraging guild framework employed by Ospina-Garcés et al. (2024), our MWBGs are based on an objective resolution of skeletal variation. Foraging guilds summarized broad ecological tendencies but grouped structurally divergent taxa within the same category (Fig. S1). For example, species classified as edge-space aerial foragers were distributed across MWBG2, MWBG3, and MWBG4, whereas edge-space trawling foragers spanned MWBG3 and MWBG5. Similarly, narrow-space gleaners were recovered across MWBG2, MWBG3, and MWBG5 (Table S1).

Within MWBG4 (*Lasiurus*), characterized by elongation of MC4, MC3, and MC2, species were differentiated into open-space and edge-space aerial foragers (Ospina-Garcés et al. 2024). In contrast, a more congruent pattern was observed within Emballonuridae: *B. plicata* and *S. bilineata* showed consistent separation across classification schemes, being assigned to MWBG2 and MWBG3 in our analysis and categorized as open- and edge-space aerial foragers by Ospina-Garcés et al. (2024). Together, these comparisons illustrate those foraging guilds and bone-based MWBGs are complementary frameworks that capture different dimensions of wing design. Importantly, MWBGs highlight intrinsic skeletal variation that is not readily captured by aerodynamic guilds and provide an additional, structure-centered perspective for investigating the ecological function of bat wings.

### Phylogeny

Our phylogenetic reconstruction, based on the time-calibrated trees of Upham et al. (2019), recovered the major chiropteran lineages in generally expected positions, with all sampled species forming a monophyletic Chiroptera except for the placement of Mormoopidae, although alternative syntheses such as the supertree of Jones et al. (2002) recover Mormoopidae as monophyletic. More recent genomic analyses (Hao et al. 2024) also support mormoopid monophyly, highlighting ongoing uncertainty in the resolution of this clade. These discrepancies underscore the challenges inherent in stabilizing higher-level relationships in bats, particularly for groups with rapid early diversification or limited fossil calibration.

Within this phylogenetic framework, the five MWBGs identified in our analyses, suggest that wing skeletal proportions in bats evolve through a combination of phylogenetic retention and iterative functional convergence. MGBW1 (Molossidae) exemplify structural conservatism, maintaining proportional configurations associated with high-speed flight (Amador et al. 2020). In contrast, MWBG2 appears in parallel within Mormoopidae and Emballonuridae, whereas MWBG3 emerges independently across three lineages: Vespertilionidae, Emballonuridae, and two separate branches within Phyllostomidae (Fig. 3). Broader comparative evidence supports this recurrence: distal element reduction and re-acquisition have independently evolved multiple times across Chiroptera (Bahlman et al. 2016), demonstrating that structurally similar architectures can arise in response to similar functional pressures. Within Phyllostomidae, nectarivorous, and frugivorous species cluster in MWBG5, suggesting diversification along a shared structural template rather than repeated independent innovation (Amador et al 2020).

### Modularity

Although our study was not designed to test for modularity explicitly, the loading patterns of the first three PCs suggest that proportional covariation in the bat wing skeleton is structured rather than random. PC1 grouped the digit 5 elements (MC5, PP5, and PD5) with distal phalanges PD4 and PD3, a configuration consistent with variation in structures related to wing chord. PC2 linked the metacarpals MC4, MC3, MC2, together with PP3, a set of elements contributing to spanwise extension and overall wing length; whereas PC3 associated MC2 with PP4 and PP5 (Table S2). These patterns support the idea that subsets of elements involved in similar aerodynamic or structural roles tend to covary, a phenomenon broadly aligned with concepts of functional-developmental modularity (Klingenberg 2008). Comparable patterns have been reported by Stevens and Guest (2022), who documented coordinated variation among metacarpals, proximal phalanges, and distal phalanges in *Ar. fimbriatus*, indicating that wing elements can form covarying units within species. The boundaries of subsets recovered here differ somewhat from those previously reported, likely reflecting the broader taxonomic and ecological scope of our dataset, but likewise suggest that the wing skeleton includes regions in which proportional traits tend to occur together.

At a macroevolutionary scale, however, Orkney et al. (2025) proposed that bat wing skeleton exhibits pervasive evolutionary integration, such that forelimb and hindlimb elements evolve as a coupled system rather than as separable modules. They further showed that different aspects of wing morphology relate to distinct ecological functions—for example, overall wing size is associated with landing maneuvers and roost access, whereas internal handwing proportions correlate with variation in flight style. Our results are consistent with this interpretation. On the one hand, some bones in our analysis loaded strongly on more than one component (notably MC2 and PP5; Table S2), indicating that individual elements can simultaneously participate in multiple coordinated trait sets, which is more consistent with integration than with discrete modularity. On the other hand, the proportional contribution of individual bones that define our MWBGs reveal that wings appearing similar in overall shape—such as those of *T. brasiliensis* (MWBG1; Fig. 1A) and *La. cinereus* (MWBG4; Fig. 1D)—can be constructed from markedly different internal proportional architectures. This suggests that, within the global structural constraint imposed by the membranous wing, internal rearrangements of skeletal proportions can generate functionally distinct solutions.

We therefore interpret this as an emergent signal of coordinated variation, rather than as formal evidence of modularity. In this view, the bat wing behaves as an integrated anatomical system that nonetheless contains recurrent subsets of elements capable of varying together. Such organization would allow evolutionary adjustments among functionally related bones without requiring wholesale restructuring of the entire wing, a hypothesis that warrants further investigation.

Overall, the patterns observed here are compatible with a scenario in which both inherited developmental factors and biomechanical demands influence the evolution of wing skeletal proportions. The mixture of conserved, convergent, and lineage-specific MWBGs highlights that proportional traits may follow different evolutionary paths and can provide additional context when compared with aerodynamic indices or foraging guilds.

## Supplementary Material

obag007_Supplemental_File

## Data Availability

The data underlying this article will be shared on reasonable request to the corresponding author.
