# Supplementary material for: Unsupervised Wing-Bone Morphogroups in Bats Reveal Phylogenetic and Functional Patterns
Source: Integr Org Biol. 2026 Mar 9;8(1):obag007. doi: 10.1093/iob/obag007 (PMC13048274; doi:10.1093/iob/obag007)
Supplement: obag007_Supplemental_File [file obag007_supplemental_file.docx]

Table S1. Average values of logarithmic ratios of 11 bone structures from the wing of Mexican bats. MWBG = Morpho-wing-bone group; n = number of individuals. Descriptive statistics: mean (min/max). *Cynmex = Cynomops mexicanus; Eumfer = Eumops ferox; Molmol = Molossus molossus, Molnig = Molossus nigricans; Nycfem = Nyctinomops femorosaccus; Nycmac = Nyctinomops macrotis; Procen = Promops centralis; Tadbra = Tadarida brasiliensis; Mormeg = Mormoops megalophylla; Pteful = Pteronotus fulvus; Ptemex = Pteronotus mexicanus; Balpli = Balantiopteryx plicata; Macwat = Macrotus waterhousii; Antpal = Antrozous pallidus; Baeall = Baeodon alleni; Baegra = Baeodon gracilis; Baudub = Bauerus dubiaquercus; Eptfus = Eptesicus fuscus; Idiphy = Idionycteris phyllotis; Myoaur = Myotis auriculus; Myocal = Myotis californicus; Myocar = Myotis carteri; Myofor = Myotis fortidens; Myothy= Myotis thysanodes; Myovel = Myotis velifer; Myovol = Myotis volans; Myoyum = Myotis yumanensis; Parhes = Parastrellus hesperus; Rhoaen = Rhogeessa aenea; Rhopar = Rhogeessa parvula; Sacbil = Saccopteryx bilineata; Desrot = Desmodus rotundus; Glysyl = Glyphonycteris sylvestris; Lascin = Lasiurus cinereus; Lasfra = Lasiurus frantzii; Lasxan = Lasiurus xanthinus; Anoper = Anoura peruana; Chogod = Choeroniscus godmani; Chomex = Choeronycteris mexicana; Glocom = Glossophaga commissarisi; Glomor = Glossophaga morenoi; Glomut = Glossophaga mutica; Hylund = Hylonycteris underwoodi; Lepyer = Leptonycteris yerbabuenae; Arthir = Artibeus hirsutus; Artjam = Artibeus jamaicensis; Artlit = Artibeus lituratus; Chisco = Chirederma scopaeum; Chivil = Chiroderma villosum; Censen = Centurio senex; Derazt = Dermanura azteca; Derpha = Dermanura phaeotis; Dertol = Dermanura tolteca; Enchar = Enchistenes hartii; Stuhon = Sturnira hondurensis; Stupar = Sturnira parvidens; Urodav = Uroderma davisi; Micmic = Micronycteris microtis; Natmex = Natalus mexicanus*. FGC = foraging guilds categories; OSAF = open space aerial foragers, ESTF = edge spaces trawling foragers, ESAF = edge spaces aerial foragers, NSGF = narrow space gleaning foragers (Ospina-Garcés et al. 2024).

|  | **FA** | **MC2** | **MC3** | **PP3** | **PD3** | **MC4** | **PP4** | **PD4** | **MC5** | **PP5** | **PD5** | **FGC** |
| --- | --- | --- | --- | --- | --- | --- | --- | --- | --- | --- | --- | --- |
| MWBG1 | | | | | | | | | | | | |
| Cynmex  n=1 | 3.56 | -0.20 | 0.06 | -0.83 | -0.79 | 0.03 | -0.98 | -2.03 | -0.39 | -1.35 | -1.98 | - |
| Eumfer  n=1 | 4.06 | -0.29 | 0.00 | -0.82 | -0.90 | 0.07 | -1.05 | -2.10 | -0.56 | -1.18 | -2.07 | - |
| Molmol  n=1 | 3.62 | -0.09 | 0.04 | -0.74 | -0.67 | 0.01 | -1.35 | -1.94 | -0.40 | -1.40 | -1.88 | - |
| Molnig  n=3 | 3.9  (3.89/  3.98) | -0.06  (-0.09/  -0.02) | 0.01  (-0.04/  0.04) | -0.79  (-0.82/  -0.78) | -0.79  (-0.86/  -0.74) | -0.02  (-0.07/  0.00) | -1.00  (-1.11/  -0.94) | -2.28  (-2.36/  -2.16) | -0.46  (-0.50/  -0.43) | -1.33  (-1.46/  -1.18) | -2.08  (-2.40/  -1.89) |  |
| Nycfem  n=4 | 3.83  (3.79/  3.88) | -0.14  (-0.23/  -0.10) | -0.03  (-0.06/  -0.01) | -0.93  (-1.00/  -0.88) | -0.71  (-0.80/  -0.66) | -0.05  (-0.09/  -0.02) | -1.20  (-1.30/  -1.13) | -2.50  (-2.74/  -2.26) | -0.63  (-0.71/  -0.55) | -1.19  (-1.24/  -1.06) | -2.30  (-2.62/  -2.09) |  |
| Nycmac  n=1 | 4.14 | -0.12 | -0.06 | -0.99 | -0.87 | -0.08 | -1.60 | -2.12 | -0.71 | -1.21 | -2.02 | - |
| Procen  n=1 | 4.00 | -0.28 | 0.05 | -0.75 | -0.79 | 0.02 | -0.91 | -2.37 | -0.42 | -1.30 | -2.10 | - |
| Tadbra  n=14 | 3.77  (3.71/  3.86) | -0.11  (-0.33/  -0.01) | -0.03  (-0.08/  0.02) | -1.08  (-1.57/  -0.97) | -0.71  (-0.77/  -0.65) | -0.06  (-0.11/  -0.01) | -1.21  (-1.27/  -1.12) | -1.68  (-1.80/  -1.49) | -0.58  (-0.66/  -0.52) | -1.25  (-1.34/  -1.18) | -1.97  (-2.20/  -1.80) | OSAF |
| MWBG2 | | | | | | | | | | | | |
| Balpli  n=1 | 3.68 | -0.17 | -0.16 | -1.42 | -1.03 | -0.37 | -1.58 | -2.41 | -0.39 | -1.42 | -2.11 | OSAF |
| Macwat  n=3 | 3.91  (3.89/  3.95) | -0.33  (-0.37/  -0.28) | -0.30  (-0.31/  -0.27) | -1.15  (-1.19/  -1.12) | -0.59  (-0.66  /-0.54) | -0.33  (-0.35/  -0.31) | -1.30  (-1.33/  -1.26) | -1.45  (-1.48/  -1.38) | -0.29  (-0.30/  -0.28) | -1.36  (-1.38/  -1.34) | -1.52  (-1.58/  -1.47) | NSGF |
| Mormeg  n=2 | 4.01  (3.99/  4.02) | -0.23  (-0.28/  -0.18) | -0.13  (-0.14/  -0.12) | -1.84  (-1.89/  -1.78) | -0.38  (-0.40/  -0.37) | -0.19  (-0.20/  -0.18) | -1.58  (-1.58/  -1.57) | -1.68  (-1.70/  -1.67) | -0.44  (-0.44/  -0.43) | -1.29  (-1.29/  -1.28) | -1.72  (-1.81/  -1.63) | ESAF |
| Pteful  n=1 | 3.73 | -0.10 | -0.04 | -1.69 | -0.53 | -0.20 | -1.72 | -1.55 | -0.28 | -1.49 | -1.43 | ESAF |
| Ptemex  n=1 | 4.04 | -0.21 | -0.22 | -1.66 | -0.56 | -0.23 | -1.69 | -1.30 | -0.25 | -1.69 | -1.48 | - |
| MWBG3 | | | | | | | | | | | | |
| Antpal  n=1 | 4.00 | -0.26 | -0.16 | -1.21 | -1.21 | -0.17 | -1.40 | -1.59 | -0.17 | -1.49 | -1.84 | NSGF |
| Beaall  n=1 | 3.34 | -0.11 | -0.07 | -0.99 | -0.60 | -0.11 | -1.25 | -1.34 | -0.08 | -1.40 | -1.91 | - |
| Baegra  n=3 | 3.48  (3.39/  3.59) | -0.06  (-0.11/  -0.02) | -0.04  (-0.08/  -0.02) | -0.94  (-1.03/  -0.88) | -0.66  (-0.72/  -0.62) | -0.05  (-0.07/  -0.03) | -1.16  (-1.27/  -1.06) | -1.26  (-1.32/  -1.23) | -0.04  (-0.06/  -0.03) | -1.30  (-1.46/  -1.13) | -1.65  (-1.70/  -1.62) |  |
| Baudub  n=1 | 3.94 | -0.05 | -0.07 | -1.45 | -0.96 | -0.09 | -1.64 | -1.48 | -0.12 | -1.98 | -2.09 | - |
| Desrot  n=1 | 4.04 | -0.14 | -0.08 | -1.79 | -0.69 | -0.09 | -1.94 | -1.35 | -0.11 | -1.88 | -1.43 | NSGF |
| Eptfus  n=3 | 3.95  (3.90/  4.01) | -0.17  (-0.19/  -0.14) | -0.11  (-0.13/  -0.09) | -1.12  (-1.14/  -1.10) | -0.76  (-0.84/  -0.68) | -0.11  (-0.12/  -0.08) | -1.21  (-1.23/  -1.19) | -1.48  (-1.52/  -1.45) | -0.15  (-0.16/  -0.13) | -1.49  (-1.53/  -1.47) | -1.87  (-1.99/  -1.72) | ESAF |
| Idiphy  n=1 | 3.76 | -0.15 | -0.08 | -1.04 | -0.87 | -0.13 | -1.25 | -1.22 | -0.37 | -1.36 | -1.70 | - |
| Myoaur  n=2 | 3.64  (3.60/  3.68) | -0.17  (-0.17/  -0.16) | -0.10  (-0.10/  -0.10) | -1.17  (-1.19/  -1.16) | -0.92  (-0.93/  -0.92) | -0.13  (-0.14/  -0.12) | -1.58  (-1.66/  -1.49) | -1.27  (-1.33/  -1.21) | -0.14  (-0.16/  -0.12) | -1.47  (-1.48/  -1.46) | -1.51  (-1.65/  -1.37) |  |
| Myocal  n=7 | 3.44  (3.35/  3.52) | -0.10  (-0.19/  -0.04) | -0.07  (-0.16/  -0.01) | -1.13  (-1.26/  -1.03) | -0.68  (-0.79/  -0.56) | -0.09  (-0.15/  -0.01) | -1.41  (-1.55/  -1.31) | -1.22  (-1.30/  -1.16) | -0.10  (-0.16/  -0.04) | -1.49  (-1.54/  -1.42) | -1.51  (-1.68/  -1.35) | ESAF |
| Myocar  n=2 | 3.51  (3.49/  3.52) | -0.18  (-0.19/  -0.17) | -0.08  (-0.09/  -0.08) | -1.27  (-1.28/  -1.26) | -0.80  (-0.81/  -0.79) | -0.11  (-0.11/  -0.10) | -1.47  (-1.51/  -1.44) | -1.35  (-1.36  /-1.34) | -0.15  (-0.16/  -0.14) | -1.72  (-1.79/  -1.65) | -1.61  (-1.73/  -1.49) |  |
| Myofor  n=1 | 3.56 | -0.11 | -0.09 | -1.20 | -0.79 | -0.08 | -1.34 | -1.26 | -0.11 | -1.51 | -1.80 | ESAF |
| Myothy  n=1 | 3.76 | -0.12 | -0.06 | -1.10 | -0.88 | -0.06 | -1.44 | -1.50 | -0.09 | -1.55 | -1.57 | - |
| Myovel  n=1 | 3.77 | -0.38 | -0.11 | -1.29 | -1.13 | -0.14 | -1.44 | -1.51 | -0.16 | -1.63 | -1.95 | ESTF |
| Myovol  n=2 | 3.31  (3.04/3.57) | -0.12  (-0.15/  -0.10) | -0.09  (-0.10/  -0.09) | -1.13  (-1.27/  -0.99) | -0.90  (-0.96/  -0.85) | -0.09  (-0.10/  -0.09) | -1.48  (-1.51/  -1.46) | -1.38  (-1.41/  -1.35) | -0.12  (-0.14/  -0.09) | -1.54  (-1.57/  -1.50) | -1.75  (-1.75/  -1.74) |  |
| Myoyum  n=2 | 3.52  (3.50/  3.55) | -0.17  (-0.24/  -0.11) | -0.16  (-0.16/  -0.15) | -1.13  (-1.18/  -1.08) | -1.22  (-1.24/  -1.20) | -0.15  (-0.17/  -0.14) | -1.30  (-1.33/  -1.27) | -1.48  (-1.48/  -1.47) | -0.18  (-0.20/  -0.15) | -1.36  (-1.41/  -1.31) | -1.65  (-1.69/  -1.61) | ESAF |
| Parhes  n=1 | 3.35 | -0.25 | -0.11 | -1.42 | -1.04 | -0.15 | -1.49 | -1.43 | -0.16 | -1.81 | -1.70 | ESAF |
| Rhoaen  n=1 | 3.30 | 0.10 | -0.01 | -0.94 | -0.48 | -0.04 | -1.16 | -1.20 | -0.02 | -1.31 | -1.93 | ESAF |
| Rhopar  n=1 | 3.35 | -0.17 | -0.12 | -1.14 | -0.63 | -0.12 | -1.32 | -1.08 | -0.14 | -1.53 | -1.58 | ESAF |
| Sacbil  n=1 | 3.79 | -0.04 | -0.01 | -1.10 | -0.80 | -0.12 | -1.58 | -1.57 | -0.16 | -1.45 | -1.67 | ESAF |
| MWBG4 | | | | | | | | | | | | |
| Lascin  n=6 | 3.97  (3.86/4.03) | 0.27  (0.22/  0.34) | 0.12  (0.03/  0.39) | -1.10  (-1.22/  -0.96) | -0.72  (-0.79/  -0.64) | -0.01  (-0.06/  0.04) | -1.56  (-1.66/  -1.47) | -1.32  (-1.37/  -1.26) | -0.23  (-0.28/  -0.16) | -1.89  (-1.97/  -1.78) | -1.70  (-1.72/  -1.68) | OSAF |
| Lasfra  n=4 | 3.69  (3.61/3.75) | 0.24  (0.21/  0.28) | 0.09  (0.08/  0.10) | -0.95  (-1.02/  -0.91) | -0.71  (-0.81/  -0.64) | 0.03  (0.02/  0.04) | -1.34  (-1.37/  -1.32) | -1.25  (-1.32/  -1.20) | -0.10  (-0.15/  -0.03) | -1.71  (-1.83/  -1.58) | -1.57  (-1.77/  -1.44) | ESAF |
| Lasxan  n=4 | 3.83  (3.79/3.88) | 0.26  (0.23/  0.30) | 0.08  (0.03/  0.12) | -1.00  (-1.04/  -0.94) | -0.85  (-0.93/  -0.82) | 0.01  (0.00/  0.02) | -1.36  (-1.40/  -1.29) | -1.47  (-1.62/  -1.30) | -0.13  (-0.15/  -0.10) | -1.82  (-1.90/  -1.75) | -1.70  (-1.74/  -1.67) | OSAF |
| MWBG5 | | | | | | | | | | | | |
| Anoper  n=1 | 3.70 | -0.11 | -0.03 | -1.09 | -0.18 | -0.06 | -1.39 | -1.15 | -0.20 | -1.61 | -1.25 | NSGF |
| Arthir  n=2 | 4.03  (4.02/4.05) | -0.19  (-0.20/  -0.18) | -0.08  (-0.10/  -0.07) | -1.32  (-1.33/  -1.30) | -0.23  (-0.24/  -0.22) | -0.07  (-0.09/  -0.04) | -1.44  (-1.46/  -1.42) | -1.08  (-1.10/  -1.06) | -0.09  (-0.09/  -0.08) | -1.76  (-1.78/  -1.73) | -1.35  (-1.35/  -1.35) | NSGF |
| Artjam  n=92 | 4.04  (3.97/4.23) | -0.15  (-0.33/  -0.07) | -0.09  (-0.17/  -0.01) | -1.27  (-1.41/  -1.04) | -0.22  (-0.39/  -0.03) | -0.10  (-0.15/  0.01) | -1.41  (-1.51/  -1.32) | -1.06  (-1.40/  -0.94) | -0.11  (-0.17/  -0.03) | -1.66  (-1.79/  -1.38) | -1.28  (-1.63/  -1.16) | NSGF |
| Artlit  n=162 | 4.19  (4.06/  4.27) | -0.15  (-0.30  -0.06) | -0.08  (-0.19/  0.01) | -1.16  (-1.36/  -0.79) | -0.19  (-0.39/  -0.05) | -0.09  (-0.20/  0.00) | -1.36  (-1.51/  -1.23) | -1.03  (-1.23/  -0.90) | -0.09  (-0.21/  -0.01) | -1.58  (-1.76/  -1.48) | -1.20  (-1.35/  -1.04) | NSGF |
| Censex  n=5 | 3.71  (3.67/  3.76) | -0.25  (-0.31/  -0.14) | -0.14  (-0.16/  -0.10) | -0.95  (-0.98/  -0.93) | -0.26  (-0.32/  -0.21) | -0.20  (-0.21/  -0.17) | -1.18  (-1.21/  -1.14) | -1.23  (-1.36/  -1.14) | -0.19  (-0.23/  -0.16) | -1.15  (-1.22/  -1.11) | -1.26  (-1.39/  -1.17) |  |
| Chisco  n=7 | 3.83  (3.80/  3.86) | -0.15  (-0.26/  0.05) | -0.03  (-0.05/  0.00) | -1.03  (-1.06/  -0.95) | -0.09  (-0.15/  -0.03) | -0.04  (-0.08/  0.01) | -1.18  (-1.23/  -1.12) | -1.05  (-1.16/  -0.98) | -0.03  (-0.09/  0.01) | -1.47  (-1.52/  -1.35) | -1.21  (-1.30/  -1.15) |  |
| Chivil  n=3 | 3.85  (3.82/  3.88) | -0.04  (-0.07/  -0.01) | 0.02  (-0.01/  0.05) | -1.02  (-1.07/  -0.97) | -0.17  (-0.19/  -0.15) | -0.01  (-0.03/  0.00) | -1.13  (-1.16/  -1.12) | -1.18  (-1.25/  -1.06) | 0.01  (-0.01/  0.02) | -1.47  (-1.55/  -1.42) | -1.22  (-1.27/  -1.17) | NSGF |
| Chogod  n=1 | 3.43 | -0.03 | 0.06 | -0.94 | -0.31 | -0.02 | -1.37 | -1.02 | -0.11 | -1.53 | -1.02 | - |
| Chomex  n=1 | 3.77 | -0.03 | 0.01 | -0.90 | -0.34 | -0.03 | -1.24 | -1.24 | -0.09 | -1.53 | -1.23 | - |
| Derazt  n=2 | 3.73  (3.70/  3.76) | -0.19  (-0.27/  -0.11) | -0.06  (-0.08/  -0.03) | -1.08  (-1.16/  -1.01) | -0.22  (-0.27/  -0.17) | -0.07  (-0.07/  -0.07) | -1.24  (-1.29/  -1.19) | -0.95  (-0.98/  -0.93) | -0.04  (-0.04/  -0.03) | -1.52  (-1.54/  -1.50) | -1.19  (-1.20/  -1.18) | NSGF |
| Derpha  n=29 | 3.60  (3.52/  3.70) | -0.13  (-0.24/  -0.06) | -0.05  (-0.12/  -0.01) | -1.08  (-1.15/  -0.99) | -0.19  (-0.28/  -0.12) | -0.06  (-0.13/  -0.02) | -1.25  (-1.37/  -1.16) | -1.04  (-1.10/  -0.94) | -0.09  (-0.14/  -0.03) | -1.46  (-1.58/  -1.37) | -1.16  (-1.29/  -1.06) | NSGF |
| Dertol  n=8 | 3.69  (3.64/  3.80) | -0.19  (-0.31/  -0.04) | -0.06  (-0.12/  -0.01) | -1.10  (-1.18/  -1.02) | -0.17  (-0.25/  -0.11) | -0.07  (-0.15/  -0.03) | -1.24  (-1.34/  -1.15) | -1.00  (-1.09/  -0.84) | -0.06  (-0.12/  -0.01) | -1.51  (-1.61/  -1.38) | -1.19  (-1.47/  -1.04) | NSGF |
| Encart  n=1 | 3.64 | -0.18 | -0.08 | -1.11 | -0.23 | -0.07 | -1.24 | -1.17 | -0.12 | -1.51 | -1.29 | - |
| Glocom  n=1 | 3.56 | -0.14 | -0.05 | -1.03 | -0.44 | -0.13 | -1.25 | -1.18 | -0.12 | -1.35 | -1.17 | - |
| Glomor  n=2 | 3.54  (3.51/  3.57) | -0.18  (-0.21/  -0.14) | -0.06  (-0.06/  -0.06) | -0.98  (-1.03/  -0.93) | -0.40  (-0.43/  -0.36) | -0.14  (-0.16/  -0.11) | -1.31  (-1.35/  -1.27) | -1.18  (-1.21/  -1.15) | -0.14  (-0.15/  -0.12) | -1.39  (-1.41/  -1.38) | -1.26  (-1.31/  -1.21) |  |
| Glomut  n=70 | 3.59  (3.50/  3.65) | -0.1  (1-0.24/  0.00) | -0.04  (-0.14/  0.03) | -1.06  (-1.17/  -0.88) | -0.38  (-0.53/  -0.28) | -0.10  (-0.14/  -0.06) | -1.33  (-1.46/  -1.25) | -1.16  (-1.29/  -1.02) | -0.18  (-0.24/  -0.11) | -1.42  (-1.59/  -1.30) | -1.21  (-1.31/  -1.12) | NSGF |
| Glysyl  n=1 | 3.73 | -0.08 | -0.07 | -1.18 | -0.46 | -0.12 | -1.46 | -1.28 | -0.05 | -1.50 | -1.61 |  |
| Hylund  n=1 | 3.44 | 0.02 | 0.12 | -0.93 | -0.30 | 0.05 | -1.31 | -1.04 | -0.04 | -1.82 | -0.92 | - |
| Lepyer  n=2 | 3.93  (3.92/  3.95) | -0.10  (-0.12/  -0.08) | -0.09  (-0.11/  -0.08) | -1.44  (-1.47/  -1.41) | -0.53  (-0.55/  -0.51) | -0.20  (-0.22/  -0.19) | -1.48  (-1.53/  -1.44) | -1.24  (-1.32/  -1.17) | -0.24  (-0.26/  -0.23) | -1.67  (-1.71/  -1.64) | -1.41  (-1.43/  -1.39) | NSGF |
| Micmic  n=1 | 3.53 | -0.22 | -0.15 | -0.94 | -0.36 | -0.14 | -1.13 | -1.31 | -0.14 | -1.22 | -1.22 | NSGF |
| Natmex  n=1 | 3.52 | -0.12 | -0.09 | -1.08 | -0.42 | -0.09 | -1.56 | -1.22 | -0.07 | -1.44 | -1.42 | ESTF |
| Stuhon  n=6 | 3.73  (3.65/  3.78) | -0.14  (-0.20/  -0.10) | -0.05  (-0.08/  -0.01) | -1.07  (-1.16/  -0.97) | -0.24  (-0.34/  -0.15) | -0.06  (-0.11/  -0.02) | -1.23  (-1.28/  -1.13) | -1.07  (-1.14/  -1.01) | -0.03  (-0.09/  0.02) | -1.55  (-1.62/  -1.34) | -1.24  (-1.31/  -1.21) | NSGF |
| Stupar  n=45 | 3.69  (3.58/  3.77) | -0.13  (-0.45/  0.06) | -0.04  (-0.13/  0.02) | -1.07  (-1.17/  -0.97) | -0.20  (-0.30/  -0.13) | -0.04  (-0.11/  0.01) | -1.28  (-1.41/  -1.14) | -1.02  (-1.19/  -0.90) | -0.06  (-0.14/  0.04) | -1.57  (-1.67/  -1.43) | -1.23  (-1.59/  -1.12) | NSGF |
| Urodav  n=1 | 3.87 | -0.34 | -0.26 | -1.32 | -0.43 | -0.31 | -1.49 | -1.27 | -0.26 | -1.74 | -1.37 | - |

Table S2. Loadings of each variable for the first three axes in PCA and LDA.

| Variable | PC1 | PC2 | PC3 | LD1 | LD2 | LD3 |
| --- | --- | --- | --- | --- | --- | --- |
| MC2 | -0.2465 | **-0.5764** | **0.6113** | -1.036 | 6.405 | 7.460 |
| MC3 | -0.3579 | **-0.784** | 0.343 | 6.840 | 2.692 | 6.526 |
| PP3 | -0.4793 | **-0.5979** | -0.4212 | -0.656 | -0.002 | 5.697 |
| PD3 | **0.7801** | -0.2745 | -0.3325 | -0.089 | -9.050 | 3.527 |
| MC4 | -0.1541 | **-0.8549** | 0.1802 | -26.246 | -5.909 | 2.356 |
| PP4 | -0.3851 | -0.4789 | **-0.6835** | -3.813 | -2.282 | 1.273 |
| PD4 | **0.8969** | -0.2942 | -0.0944 | 0.900 | 2.432 | 1.667 |
| MC5 | **0.7886** | -0.3967 | 0.017 | 21.983 | 9.381 | -8.330 |
| PP5 | **-0.5883** | -0.0112 | **-0.6647** | 3.059 | -2.434 | -7.167 |
| PD5 | **0.8414** | -0.2867 | -0.2665 | 4.627 | -1.573 | 0.708 |

Table S3. Discriminant analysis classification matrix using cross validation (LOOCV): A = by Morpho-wing bone groups (MWBG); B = by foraging-guilds. OSAF = open space aerial foragers, ESTF = edge spaces trawling foragers, ESAF = edge spaces aerial foragers, NSGF = narrow space gleaning foragers.

| A | Predicted groups | | | | | |
| --- | --- | --- | --- | --- | --- | --- |
| Observed groups | MWBG1 | MWBG2 | MWBG3 | MWGG4 | MWBG5 | % |
| MWBG1 | 26 | 0 | 0 | 0 | 0 | 100 |
| MWBG2 | 0 | 6 | 1 | 0 | 1 | 75 |
| MWBG3 | 0 | 1 | 28 | 0 | 5 | 88.24 |
| MWBG4 | 0 | 0 | 0 | 14 | 0 | 100 |
| MWBG5 | 0 | 0 | 4 | 0 | 440 | 99 |
| B | ESAF | ESTF | - | NSGF | OSAF | % |
| ESAF | 16 | 1 | 1 | 4 | 2 | 66.67 |
| ESTF | 1 | 0 | 0 | 1 | 0 | 0 |
| - | 11 | 2 | 9 | 18 | 4 | 20.45 |
| NSGF | 1 | 1 | 1 | 428 | 0 | 99.3 |
| OSAF | 1 | 0 | 1 | 0 | 23 | 92 |


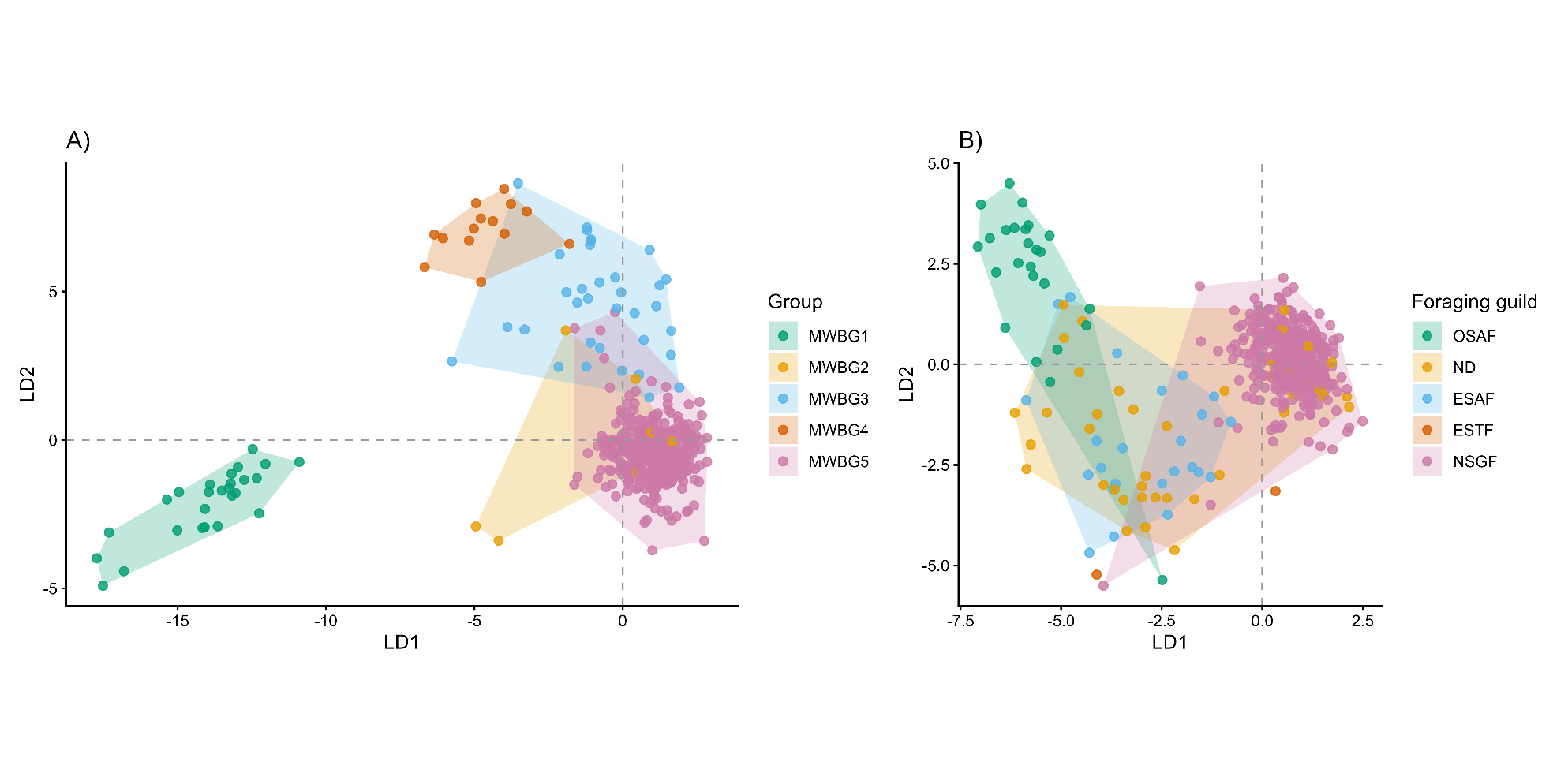


Figure S1. Linear discriminant analysis (LDA) morphospace including 59 species and 526 individuals. (A) Distribution of individuals according to the resulting morpho–wing bone groups (MWBGs). (B) Distribution based on the foraging guild categories of Ospina-Garcés et al. (2024): OSAF = open-space aerial foragers; ESTF = edge-space trawling foragers; ESAF = edge-space aerial foragers; NSGF = narrow-space gleaning foragers; ND = no assigned category.
